# Asymmetric somatic hybridization induces point mutations and indels in wheat

**DOI:** 10.1186/s12864-015-1974-6

**Published:** 2015-10-17

**Authors:** Mengcheng Wang, Chun Liu, Tian Xing, Yanxia Wang, Guangmin Xia

**Affiliations:** The Key Laboratory of Plant Cell Engineering and Germplasm Innovation, Ministry of Education, School of Life Science, Shandong University, 27 Shandanan Road, Jinan, 250100 Shandong P. R. China; Shijiazhuang Academy of Agriculture and Forestry Sciences, 479 Shengli North Avenue, Shijiazhuang, 050041 Hebei China

**Keywords:** Introgression line, Allopolyploidy, Single nucleotide polymorphism, Insertion, Deletion, Genetic variation

## Abstract

**Background:**

Allopolyploid genome needs wide structural variation to deal with genomic shock. The introgression line, generated via asymmetric somatic hybridization, is introgressed with a minimum of exogenous chromatin, which also leads to genomic shock to induce genetic variation. However, the extent of its genomic variation and its difference from allopolyploidies remains unknown.

**Methods:**

Here, we explored this issue using the bread wheat cultivar SR3, a derivative of an asymmetric somatic hybrid between the cultivar JN177 and an accession of tall wheatgrass (*Thinopyrum elongatum*). The ESTs (expressed sequence taqs) were large-scale sequenced using the cDNA library constructed in each of SR3 and JN177. Point mutations and indels (insertions and deletions) of SR3 were calculated, and their difference from the genetic variation of bread wheat and its ancestors were compared, with aim to analyze the extent and pattern of sequence variation induced by somatic hybridization.

**Results:**

Both point mutations and indels (insertions and deletions) were frequently induced by somatic hybridization in the coding sequences. While the genomic shock caused by allopolyploidization tends to favor deletion over insertion, there was no evidence for such a preference following asymmetric somatic hybridization. The GC content of sequence adjacent to indel sites was also distinct from what has been observed in allopolyploids.

**Conclusions:**

This study demonstrates that asymmetric somatic hybridization induces high frequency of genetic variation in a manner partially different from allopolipoidization. Asymmetric somatic hybridization provides appropriate material to comprehensively explore the nature of the genetic variation induced by genomic shock.

**Electronic supplementary material:**

The online version of this article (doi:10.1186/s12864-015-1974-6) contains supplementary material, which is available to authorized users.

## Background

Anthropogenic selection applied during the domestication and improvement of a crop species inevitably reduces its genetic base, while its non-domesticated wild relatives retain genetic diversity. The wild relatives thus represent a valuable genetic resource for breeders. Introgressing genetic material from a wild species is conventionally attempted via sexual hybridization [1, 2], but somatic hybridization (the induced fusion of somatic cells, followed by their *in vitro* regeneration) can offer an alternative route, especially where viable sexual hybrids have proven difficult or impossible to establish [3]. Unlike in an allopolyploid, where the two (or more) parental nuclear genomes are combined with a uniparental cytoplasm (that of the female parent of the hybrid), in a somatic hybrid, both the nucleus and the cytoplasm are biparental. In a refinement of the approach, termed asymmetric hybridization, the donor cells are irradiated prior to the fusion process, which has the effect of reducing the contribution of the donor’s genome to the somatic hybrid, thereby promoting the introgression of smaller donor segments [4, 5].

Bread wheat (*Triticum aestivum*) is an allohexaploid species which has evolved through two successive natural hybridizations. The first brought together the genomes of an as yet unidentified (possibly extinct) member of the *Sitopsis* section of the genus *Aegilops* (B genome) and *T. urartu* (A genome) to form the allotetraploid BA genome species *T. turgidum* [6]. The second event involved a domesticated form of *T. turgidum* and *Ae. tauschii* (D genome) to form the bread wheat BAD genome [6]. Allopolyploidy, in forcing a pair of non-identical genomes to co-exist within a single nucleus, has been associated with a range of genomic rearrangements and DNA modifications [7, 8], encapsulated by the term “genomic shock” [9]. Some of these changes affect gene expression, either by altering gene sequences and/or by altering their epigenetic regulation [9–20], thereby providing the polyploid genome with extensive potential for novelty and plasticity [20–23]. Once a wide hybrid (whether sexual or somatic) has been established, introgression from one parental genome to the other can occur via recombination or meiosis-driven chromosome breakage and reunion. In an asymmetric somatic hybrid, the pre-hybridization irradiation treatment of the donor cells has the effect of fragmenting its genome, so introgression can occur via end-joining of fragments, most easily during the course of mitosis [24]. This event also leads to a strong genomic shock and therefore induces genomic variation. However, in asymmetric somatic fused cells, most donor chromatin is eliminated, and very small amount of chromatin fragments are introgressed into the recipient genome. Thus, chromosomal rearrangement and large fragment deletion, the common events during the process of diploidization of allopolyploidies, seldom happen in asymmetric somatic hybrid cells [3]. This difference between the chromosomal behaviors in asymmetric somatic hybrids and allopolyploidies suggest that their patterns of genomic variation may be distinct from each other.

We previously generated an asymmetric somatic hybrid between the bread wheat cultivar JN177 (with modest salt tolerance) and tall wheatgrass (*Thinopyrum elongatum*) (wheat’s close relative, one of the monocots with highest salt tolerance) via fusing the wheat protoplast and UV-irradiated tall wheatgrass protoplast (chromatin was fragmented), and a number of derivatives of this hybrid have been selected based on the expression of a favorable phenotype [3, 5, 25, 26]. Some of these lines have proven to be stably introgressed with several (5 ~ 7) observed chromatin fragments of tall wheatgrass by cytogenetic analysis [27], and be sufficiently phenotypically stable to allow their release as a novel cultivar [25, 26]. One of these is the cultivar SR3 whose genome possesses five observed fragments of tall wheatgrass [27]. It has demonstrated improved levels of tolerance to both salinity and drought, and higher yield in saline land than its parent JN177 [28, 29]; the tolerance is partially due to the high tolerance to oxidative stress and superior ability of redox homeostasis maintenance [28, 30]. We found that the genome of SR3 and other derivatives occurred high frequency of genetic variation using a set of molecular biomarker assays [31], and had numerous single nucleotide polymorphisms (SNPs) and insertion/deletion (indels) when comparing genomic sequences of glutenin gene family [32]. Moreover, such genetic variation was majorly induced by asymmetric somatic hybridization, while the contribution of other factors such as protoplast formation, UV radiation, callus induction and plant regeneration was quite slight [31, 32]. However, the characteristics of sequence variation such as nucleotide substitution and indel at the genome-scale level as well as its difference from polyploidization have not been addressed. Although contrasting allelic sequences have been identified between SR3 and JN177 associated mostly with genes involved in the response to abiotic stress [30, 33, 34], a more global picture of the alterations induced by asymmetric somatic hybridization awaits the application of genome-wide re-sequencing and transcriptomic analysis. While global genomic re-sequencing remains overambitious given the large size of the wheat genome [35] along with the high content of repeat sequences (>80 %) [36], current DNA sequencing platforms are well capable of uncovering sequence variation in the transcriptome. Here, the sequencing of a large number of cDNAs isolated from both SR3 and JN177 is described and used to show the extent of the sequence variation induced by asymmetric somatic hybridization.

## Results

### cDNA sequencing

A total of 19,045 SR3 and 10,327 JN177 clones were sequenced, resulting in the acquisition of, respectively, 18,192 and 9770 usable sequences (Additional file 1: Table S1). The sequences resolved into 9634 unigenes (2097 contigs and 7537 singletons) from SR3, and 7107 unigenes (1207 contigs and 5900 singletons) from JN177, of which full length cDNAs were 4825 and 2975, respectively (Additional file 1: Table S1). The length of most of the unigenes laid in the range 700–1000 nt (Additional file 2: Figure S1), and their mean GC content was 53.85 % (SR3) and 55.46 % (JN177). The BLASTn analysis of the unigenes revealed that 2581 were shared (>96 % identity) between SR3 and JN177 (Table 1). In all, 5072 (71.4 %) of the JN177 and 7284 (75.6 %) of the SR3 unigenes shared >96 % identity with sequences represented in the wheat EST database (Table 1).

**Table 1:** BLASTn-based homology comparisons of unigene sequences

Alignment	Total unigenes	Matched unigenes		
		Total	>96 % identity	>96 % identity coverage (%)
SR3-JN177	9635	4677	2581	75.25
SR3-Ta	9635	9097	7284	85.22
JN177-Ta	7107	6466	5072	84.14

SR3-JN177: JN177 unigene sequences queried with those of SR3. SR3-Ta: SR3 unigene sequences queried with wheat ESTs housed in GenBank. JN177-Ta: JN177 unigene sequences queried with wheat ESTs housed in GenBank

### Frequency of single nucleotide polymorphisms (SNPs) in the unigene sequences

Based on the unigene sequences sharing >96 % identity, 15,226 SNPs were identified within the unigene sequence shared between SR3 and JN177, equivalent to a SNP frequency of 11.33 per 1000 nt of coding sequence (Table 2). The transition and transversion frequencies were, respectively, 6.70 and 4.63 per 1000 nt. The SNP frequency between JN177 and the sequences represented in the wheat EST database (JN177 *vs* Ta comparison) was only about one half of this level (5.77 per 1000 nt) (Table 2), demonstrating that the somatic hybridization process was effective in inducing point mutations. A comparison based on the sequences of the unigenes shared between the BA progenitor tetraploid (*T. turgidum*) and the A genome carrier *T. monococcum* revealed a SNP frequency of 15.48 per 1000 nt, while that between *T. turgidum* and *Ae. speltoides* (related to the B genome progenitor) was 18.51, indicating that a high frequency of mutation was induced during the formation of allotetraploid wheat. Similarly, the estimated SNP frequencies between bread wheat and *T. monococcum*, *Ae. speltoides*, *T. turgidum* and *Ae. tauschii* (D genome progenitor) were, respectively, 12.02, 16.24, 12.13 and 5.40 per 1000 nt (Table 3). Thus the mutation frequency induced by the somatic hybridization process appeared to be similar in extent to that induced by allopolyploidization. The frequency of SNPs between the unigene sequences of bread wheat and those of either *T. monococcum* or *Ae. speltoides* was less than that between *T. turgidum* and either *T. monococcum* or *Ae. speltoides* unigenes (Table 3). This coincided with the finding that the SNP frequency of SR3 and wheat database EST (SR3 *vs* Ta alignment) was lower than those of the SR3 *vs* JN177 alignment (Table 2). The SNP frequency between SR3 unigene sequences and those of the A, B, BA and D genome species was similar to those between JN177 unigenes and those of the A, B, BA and D genome species (Table 3).

**Table 2:** The SNP frequencies in SR3 and JN177

SNPs	Type	SNPs (per 1000 nt)		
		SR3-JN177	SR3-Ta	JN177-Ta
Transitions	C → T	2.065	0.788	0.727
	T → C	1.776	1.016	1.031
	G → A	1.542	1.168	0.843
	A → G	1.318	0.641	0.602
	Total	6.701	3.613	3.203
Transversions	A → T	0.267	0.154	0.139
	T → A	0.252	0.208	0.180
	C → G	1.142	0.399	0.412
	G → C	1.084	0.680	0.671
	G → T	0.455	0.320	0.297
	T → G	0.386	0.273	0.281
	A → C	0.507	0.243	0.239
	C → A	0.533	0.349	0.342
	Total	4.627	2.625	2.561
Total SNPs		11.328	6.238	5.764

SR3-JN177: JN177 unigene sequences queried with those of SR3. SR3-Ta: SR3 unigene sequences queried with wheat ESTs housed in GenBank. JN177-Ta: JN177 unigene sequences queried with wheat ESTs housed in GenBank

**Table 3:** SNP and indel frequencies among wheat and its ancestors

Query	Subject	SNPs (per 1000 nt)			Indels (per 1000 nt)		
		Transition	Transversion	Total	Insertion	Deletion	Total
*Triticum turgidum*	*Triticum monococcum*	9.436	6.048	15.484	0.622	1.282	1.904
	*Aegilops speltoides*	10.415	8.091	18.506	0.565	0.717	1.282
*Triticum aestivum*	*Triticum monococcum*	7.258	4.762	12.020	0.688	0.769	1.457
	*Aegilops speltoides*	9.709	6.531	16.240	0.810	0.606	1.417
	*Triticum turgidum*	7.096	5.036	12.131	1.259	0.755	2.014
	*Aegilops tauschii*	4.368	1.028	5.396	0.257	2.055	2.312
JN177	*Triticum monococcum*	9.848	6.395	16.243	0.910	0.473	1.383
	*Aegilops speltoides*	9.631	7.060	16.692	0.725	0.526	1.251
	*Triticum turgidum*	7.299	6.113	13.412	1.252	0.348	1.601
	*Aegilops tauschii*	5.010	1.474	6.484	0.000	4.421	4.421
SR3	*Triticum monococcum*	10.053	6.358	16.411	1.319	0.554	1.873
	*Aegilops speltoides*	10.654	6.931	17.585	1.141	0.581	1.722
	*Triticum turgidum*	7.709	5.352	13.060	1.480	0.469	1.949
	*Aegilops tauschii*	9.123	1.610	10.733	0.716	4.651	5.367
	*Triticum aestivum*	3.613	2.625	6.238	1.047	0.307	1.354

### The size distribution of indels in the unigene sequences

**Fig. 1:**
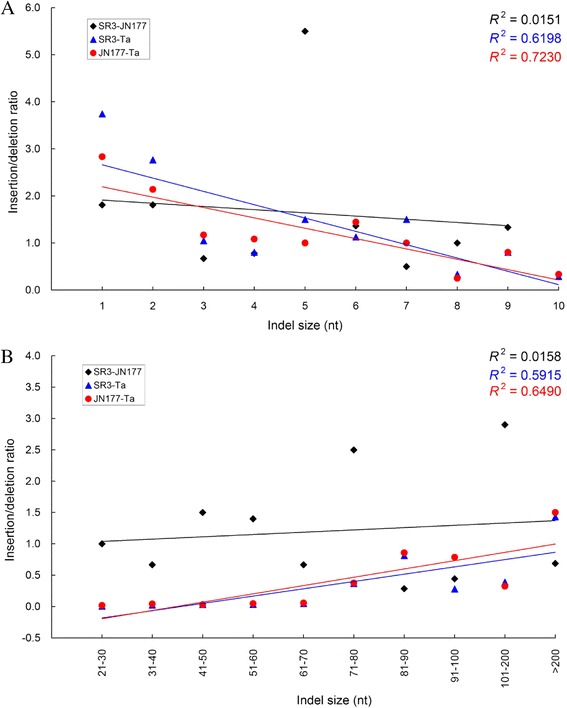
The distribution of indel lengths. **a**: small indels. **b**: large indels. The insertion/deletion ratio was obtained by dividing the number of insertions by the number of deletions. SR3-JN177: JN177 unigene sequences queried with those of SR3. SR3-Ta: SR3 unigene sequences queried with wheat ESTs housed in GenBank. JN177-Ta: JN177 unigene sequences queried with wheat ESTs housed in GenBank. The correlation between indel size and insertion/deletion ratio was performed using the Pearson correlation analysis

The indels ranged from 1 nt to 574 nt, and a majority of the indels involved only 1 nt. A significant number of indels was revealed by aligning the matched unigene sequences, with the frequency of larger indels (>23 nt) being clearly less than that of the small ones (1–10 nt) (Table 4). There had 82.14 % unigenes possessing small indels when compared between SR3 and JN177, higher than those from SR3 *vs* Ta and JN177 *vs* Ta comparisons. On the contrary, 6.70 % unigenes had large indels in the comparison between SR3 and JN177, lower than those of other two comparisons. There had more unigenes with small insertions than those with small deletions, and the difference was stronger in the SR3 *vs* Ta and JN177 *vs* Ta comparisons. Unigenes with large insertions were similar to those with large deletions in the SR3 *vs* JN177 comparison, but unigenes with large deletions were more abundant than those with large insertions in the other two comparisons. The comparison between the JN177 (and similarly SR3) unigene sequences with those represented in the wheat EST database showed that for small indels, the ratio of insertion to deletion frequency was negatively correlated to indel length (*R*^2^ = 0.62 and 0.72, respectively) (Fig. 1a); the ratio was >1 for indels shorter than 6 nt, and <1 for indels longer than 6 nt. However, for the larger indels, the insertion to deletion ratio was positively correlated to indel length (*R*^2^ = 0.59 and 0.65, respectively) (Fig. 1b); in indels ranging in length from 20 to 70 nt, the ratio was just 0.01–0.06, rising to 0.28–0.86 for indels of length 71–200 nt, and to ~1.5 for indels longer than 200 nt (Fig. 1b). The SR3 *vs* JN177 comparison revealed an insertion to deletion ratio of ~1 irrespective of indel length (Fig. 1a, b).

**Table 4:** Indel variation in SR3 and JN177 unigene sequences

Sizes	Type	SR3-177 (%)	SR3-TA (%)	177-Ta (%)
Small indels	Insertion	51.57	58.02	37.66
(1–10 nt)	Deletion	30.57	17.02	14.57
	Indel	82.14	75.04	52.23
Large indels	Insertion	3.45	1.75	1.32
(23–574 nt)	Deletion	3.25	9.8	8.94
	Indel	6.70	11.55	10.26

“Small” indels were defined as those lying within an aligned sequence, while “large” indels split matched sequence into two separate alignments. The table shows the percentage of unigenes affected by insertion/deletions. SR3-JN177: JN177 unigene sequences queried with those of SR3. SR3-Ta: SR3 unigene sequences queried with wheat ESTs housed in GenBank. JN177-Ta: JN177 unigene sequences queried with wheat ESTs housed in GenBank

### The frequency of indels in the unigene sequences

Small indels were used to calculate the indel frequency because they were markedly more abundant than large indels (indel numbers not shown). In all, the 2581 matched unigenes derived from the SR3 *vs* JN177 comparison revealed 2120 indels (1.58 per 1000 nt). Based on the JN177 sequences, these comprised 1331 insertions and 789 deletions in SR3, equivalent to frequencies of, respectively, 0.99 and 0.59 per 1000 nt (Table 5). In the comparison with the sequences represented in the wheat EST database, the indel frequency in SR3 was 1.36 per 1000 nt. The similar comparison between JN177 and the sequences represented in the wheat EST database revealed an indel frequency of only 0.93 per 1000 nt, implying that the asymmetric somatic hybridization process was effective in inducing indels in coding sequence.

**Table 5:** The frequency of small indels in SR3 and JN177 unigene sequences

Length (nt)	SR3-177 (per 1000 nt)	SR3-TA (per 1000 nt)	177-Ta (per 1000 nt)
1	1.319	1.229	0.823
2	0.109	0.063	0.048
3	0.076	0.032	0.031
4	0.031	0.009	0.009
5	0.010	0.006	0.004
6	0.019	0.008	0.008
7	0.004	0.001	0.002
8	0.003	0.001	0.004
9	0.005	0.002	0.003
10	0.001	0.002	0.001
Insertions	0.990	1.047	0.672
Deletion	0.587	0.307	0.260
Indels	1.577	1.354	0.932

“Small” and “large” indels defined as in Table 4. Indel frequency was expressed as the number of indels per 1000 nt of unigene sequence

To compare the induction rates of indels caused by somatic hybridization and allopolyploidization, equivalent calculations were made using the matched unigene sequences present in bread wheat and its relatives/progenitor species (Table 3). Comparing the unigenes of *T. turgidum* with those of *T. monococcum* and *Ae. speltoides* revealed an indel frequency of, respectively, 1.90 and 1.28 per 1000; the equivalents between bread wheat and its various related species ranged from 1.42 to 2.31 per 1000 nt, with the comparison involving *Ae. tauschii* producing the highest estimate. The indel frequencies estimated using the unigenes of JN177 and SR3 also lay in the range 1 to 2 per 1000 nt, with the exception of the comparison with *Ae. tauschii*, where the frequencies were, respectively, 4.42 and 5.37 per 1000 nt (Table 3).

In the SR3 *vs* JN177 comparison, the insertion frequency (0.99 per 1000 nt) was 1.69 fold to the deletion frequency (0.59 per 1000 nt) (Table 5). Based on the sequences represented in the wheat EST database, the insertion frequencies were higher by 3.41 and 2.58 fold than deletion frequencies in SR3 and JN177, respectively. Especially, the insertion frequency in the SR3 *vs* Ta comparison (1.05 per 1000 nt) was similar to the SR3 *vs* JN177 comparison (0.99 per 1000 nt), but its indel frequency was lower than the latter. Thus, the preference to small insertion was decreased in wheat asymmetric somatic hybrids in comparison with allopolyploid wheat.

### The function of unigenes showing sequence polymorphism

To know whether the genetic variation is associated with their biological processes, we selected sequences participating in gene expression regulation and other processes for analysis. A selection of polymorphic unigenes represented in the JN177 and SR3 libraries (948 and 1519, respectively) as well as in the wheat EST database showed that for gene expression regulation, the frequency of SNPs differed most notably in genes involved in nucleosome assembly and chromatin assembly/disassembly (Table 6). The least polymorphic comparison was between JN177 and the wheat EST database unigenes (4.59 SNPs per 1000 nt for the genes involved in the former category and 3.72 per 100 nt in the latter). The same comparison between SR3 and JN177 produced, respectively, 18.17 and 19.21 SNPs per 1000 nt. The next most variable genes were those encoding products involved in translation and post-translational modification, where the SR3 *vs* JN177 comparison revealed a SNP frequency of, respectively, 13.30 and 13.97 per 1000 nt, while the JN177 *vs* Ta comparison showed 8.69 and 7.98, respectively. The range in SNP frequency for genes associated with metabolic processes ranged from 5.45 to 6.29 per 1000 nt in the JN177 *vs* Ta comparison and 9.16–14.25 per 1000 nt in the SR3 *vs* JN177 comparison (Additional file 3: Table S2). SNP frequencies were also higher in the SR3 *vs* Ta comparison than in the JN177 *vs* Ta comparison for genes encoding proteins involved in various metabolic processes except for glycolysis as well as nucleobase, nucleoside, nucleotide and nucleic acid metabolic process (Additional file 3: Table S2). This difference in SNP frequencies was also found in ESTs of protein fate, transport, cell redox homeostasis, and response to (oxidative) stress (Additional file 4: Table S3). With respect to unigenes varying at the level of indels, the frequency of polymorphism was lower in the JN177 *vs* Ta than in the SR3 *vs* JN177 comparison (Table 6; Additional files 3 and 4: Table S2 and S3). Indel events were noticeably rare in genes involved in nucleosome assembly and chromatin assembly/disassembly (Table 6). The respective frequencies were 0.43 and 0.38 per 1000 nt in the JN177 *vs* Ta comparison and 0.95 and 0.92 per 1000 nt in the SR3 *vs* JN177 comparison.

**Table 6:** The function of unigenes affected by SNP and indels in SR3 and JN177

Process	Unigene number		SNP (per 1000 nt)			InDel (per 1000 nt)		
	JN177	SR3	SR3-JN177	SR3-Ta	JN177-Ta	SR3-JN177	SR3-Ta	JN177-Ta
Chromatin assembly or disassembly	54	92	18.17	7.07	4.59	0.95	0.85	0.43
Nucleosome assembly	49	83	19.21	6.8	3.72	0.92	0.75	0.38
Regulation of transcription	96	204	10.79	9.85	11.49	1.94	1.53	1.22
RNA processing	35	85	10.39	10.65	9.73	1.94	1.33	1.02
Translation	254	373	13.30	8.77	8.69	1.71	1.40	1.08
Protein modification process	266	440	11.39	10.61	10.19	1.87	1.46	1.32
Protein phosphorylation	181	230	10.81	12.08	11.7	2.09	1.53	1.41
Post-translational protein modification	13	12	13.97	9.92	7.98	1.28	1.23	1.00

SR3-JN177: JN177 unigene sequences queried with those of SR3. SR3-Ta: SR3 unigene sequences queried with wheat ESTs housed in GenBank. JN177-Ta: JN177 unigene sequences queried with wheat ESTs housed in GenBank

### Sequences flanking indels in the unigenes

**Fig. 2:**
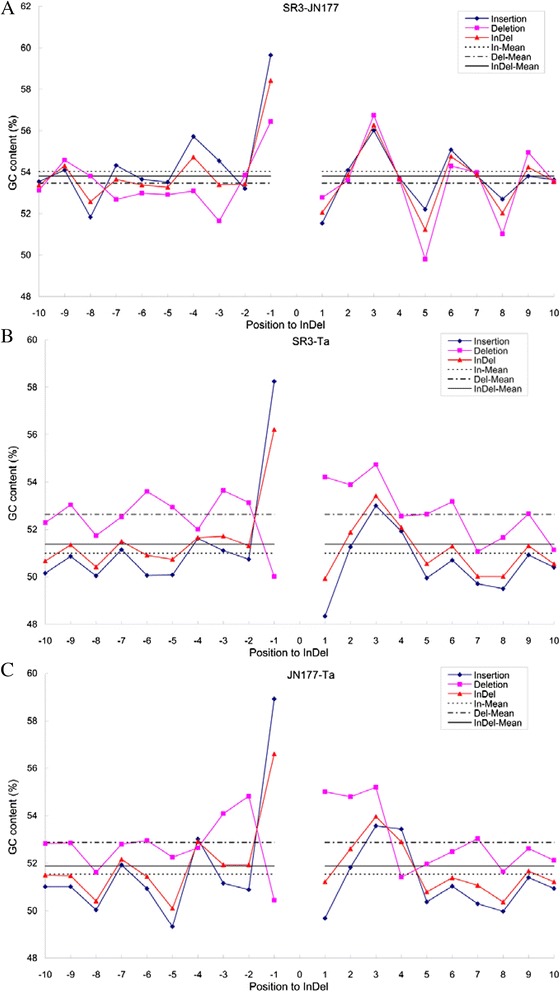
Variation in the GC content in the sequence immediately flanking indels. **a**: SR3-JN177, JN177 unigene sequences queried with those of SR3. **b**: SR3-Ta, SR3 unigene sequences queried with wheat ESTs housed in GenBank. **c**: JN177-Ta, JN177 unigene sequences queried with wheat ESTs housed in GenBank. -10 ~ −1: The tenth to first nucleotides on the 5′ side of the indel. 1 ~ 10: The first to tenth nucleotides on the 3′ side of the indel. In-mean, Del-mean and indel-mean: the GC content of 5′ and 3′ flanking sequences of insertions, deletions and indels, respectively

The sequences flanking indels were characterized by calculating the GC content of the ten nucleotides flanking either side of the indel. There was no obvious difference in GC content between 5′ and 3′ terminal flanking sequence in any of the comparisons (JN177 or SR3 *vs* wheat database ESTs, SR3 *vs* JN177) (data not shown). The trend of GC content was similar when the second to tenth nucleotides of the flanking sequence were considered (Fig. 2). The GC content of the nucleotides positioned three, six and nine away from the indels was higher than that of the ones positioned four, five, seven or eight away in the 3′ terminal flanking sequences, but the rule was not found in the 5′ terminal flanking sequences. The GC content of the flanking sequence was higher in the SR3 *vs* JN177 comparison (53.47–54.04 %) than in the other two comparisons (51.88–52.88 % in JN177 *vs* Ta, 50.99–52.63 % in SR3 *vs* Ta). In the SR3 *vs* Ta and JN177 *vs* Ta comparisons, the GC content of the flanking sequence of the deletions was higher than that of the insertions, while the content of the indels was close to that of the insertions. However, in the SR3 *vs* JN177 comparison, the GC content of the flanking sequence of the deletions was generally lower than that of the insertions, and the difference between the deletions and insertions was weaker than that in the JN177 or SR3 *vs* Ta comparisons; the content of the flanking sequence of the indels did not bias toward either the deletions or insertions. In the JN177 or SR3 *vs* Ta comparisons, with respect to the nucleotide lying on the 5′ side of the indels, the GC content of the nucleotide adjacent to the deletions was significantly lower than that of other nucleotides in the flanking sequence, but the GC content of the nucleotide adjacent to the insertions was significantly higher than that of other nucleotides in the flanking sequence (Fig. 2b, c). On the other hand, for the nucleotides lying on the 3′ side of the indels, the GC content of the nucleotide adjacent to the deletions remained high and was comparable to that of the second and third flanking nucleotides, but the GC content of the nucleotide adjacent to the insertions was significantly lower than that of the other flanking nucleotides (Fig. 2b, c). In contrast in the SR3 *vs* JN177 comparison, for the nucleotides lying on the 5′ side of the indel, the GC content of the nucleotide adjacent to both the deletions and insertions was higher than that of the second to tenth nucleotides of the 5′ flanking sequence, while for the nucleotides lying on the 3′ side of the indels, the GC content of the nucleotide adjacent to both the deletions and insertions was lower than the second and third nucleotides (Fig. 2a).

### Indel classification

**Fig. 3:**
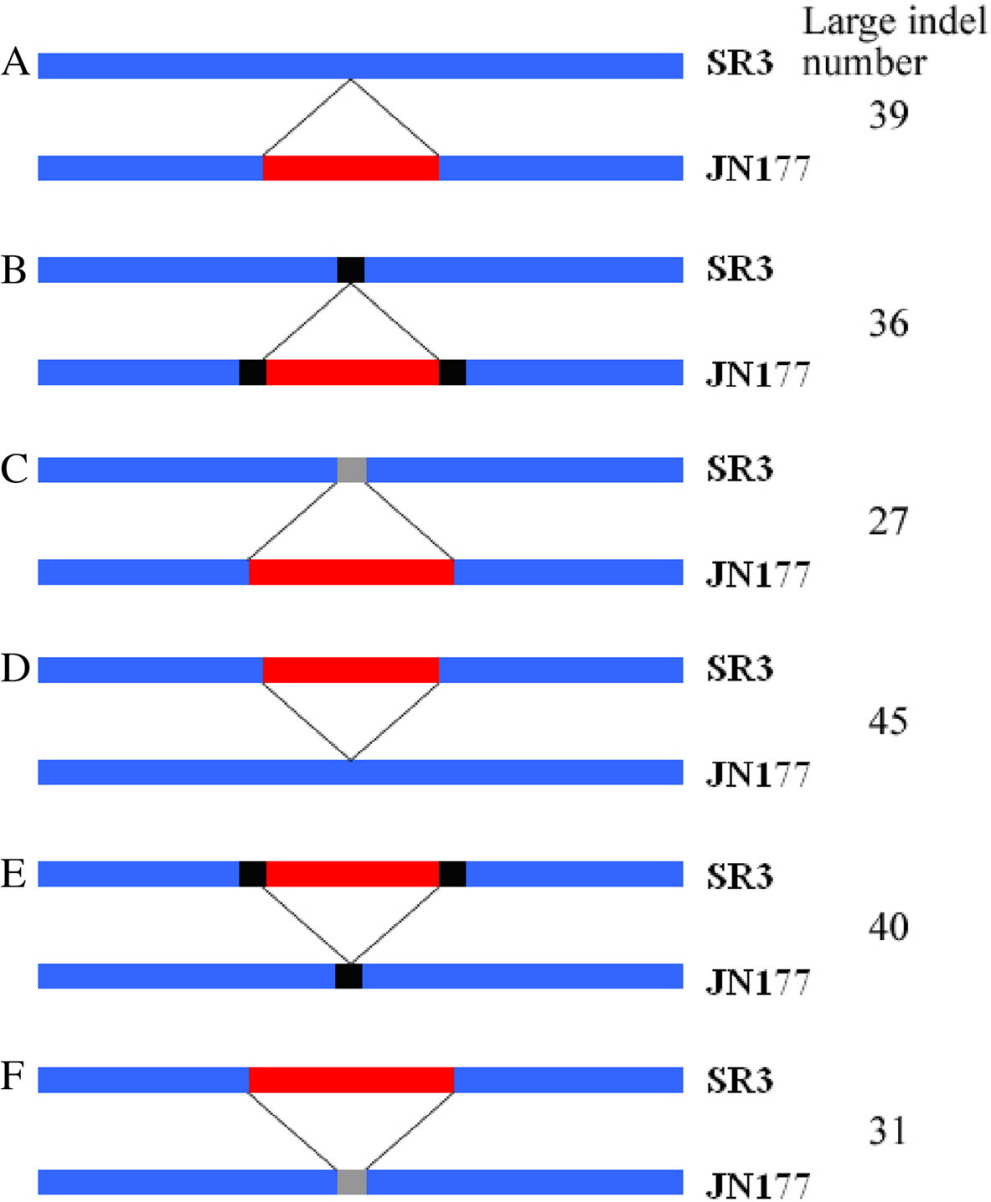
Hypothetical model for the formation of large indels induced during asymmetric somatic hybridization. Blue block: unigenes shared by SR3 and JN177. Red block: insertion and deletion fragments. Black block: repetitive sequences in the indel flanking sequence. Gray blocks: small fragments adjacent to insertions and deletions, differing sequence between SR3 and JN177. SR3-JN177: JN177 unigene sequences queried with those of SR3. SR3-Ta: SR3 unigene sequences queried with wheat ESTs housed in GenBank. JN177-Ta: JN177 unigene sequences queried with wheat ESTs housed in GenBank

We further compared the characteristic of flanking sequences of large indels in the SR3 *vs* JN177 comparison. The flanking sequences of 45 large insertions and 39 large deletions identified in the SR3 *vs* JN177 comparison were identical (Fig. 3a, d). Two examples were SR3_2LCP226_E06 (474 nt insertion) and SR3_firstas1573 (34 nt deletion) (Additional file 5: Figure S2A, B). A second group had 40 insertions and 36 deletions, whose two terminals possessed repeated sequences in SR3 and JN177 (Fig. 3b, e). The flanking sequence of SR3_5V50 (179 nt insertion) harbors a run of G’s; the 3′ flanking sequence of SR3_firstas843 (55 nt deletion) includes two copies of CATCCC in JN177 but only one in SR3 (Additional file 5: Figure S2C, D). The repeat motifs present in the flanking sequence ranged in length from 1 to 51 nt (data not shown). A 1 nt motif was present in 23 of the insertions and 19 of the deletions, dominated by runs of G (data not shown). The third group, in which the flanking sequence was modified (Fig. 3c, f), is exemplified by SR3_firstas1573 (141 nt deletion), where SNPs were generated at three positions adjacent to the deletion (Additional file 5: Figure S2E). A few of the unigenes experienced multiple indel events: SR3_firstas1573 carries two deletions, one belonging to the first group and the other to the third group (Additional file 5: Figure S2B, E). Other variants included the induction of translocated and chimeric sequences. In the homologs SR3_2LCP192_G10 and JN177_firstas231, the same sequence was found in positions 1187–1367 in the former allele, but at 1–181 in the other. SR3_2LCP192_G10 also harbors a large deletion with a repeat sequence CAAGAAGGA (Additional file 6: Figure S3A). SR3_firstas716 nucleotides 88–196 do not align with JN177_LCP139_D11 nucleotides 157–278, but their terminal sequences are identical (Additional file 6: Figure S3B).

## Discussion

### Asymmetric somatic hybridization induces sequence variation

A manifestation of genomic shock, which has been experimentally observed in *de novo* wide hybrids and inferred from the analysis of natural allopolyploids [37], is a widespread alteration of DNA sequence in the form of both point mutations and indels [38–40]. The alternation was found confirmed by the comparison between bread wheat and its ancestors (Table 3) as well as between SR3/JN177 and sequences of wheat EST database (Table 2,5). The present comparison between the transcriptomes of SR3 and JN177 has shown that the somatic hybridization process also perturbs the genome in this way (Table 2,5). AFLP fingerprinting has also suggested that non-genic sequence also appear to suffer mutation [31]. Cytogenetically and Phenotypically, SR3 (as well as other derivatives of wheat-based somatic hybrids) has been shown to be highly stable [5, 25, 26], which implies that genetic instability prevails only in the immediate aftermath of the wheat somatic hybridization event, at least in the short term.

The epigenetic modification of genic sequences is a common feature of allopolyploid genomes [12, 13, 41, 42]. The proportion of loci experiencing an alteration in cytosine methylation in a newly synthesized allohexaploid wheat has been estimated to be as high as 13 % [12]. As methylated cytosines, especially those present as a CpG dinucleotide, are readily converted to thymine [43], so - as demonstrated in *Arabidopsis thaliana -* DNA methylation can represent a major source of SNP formation [44]. In plant genomes, the bias of synonymous codon usage towards thymine and adenine is also driven by DNA methylation [45]. Both somatic hybridization and allopolyploidization result in change in cytosine methylation [12, 41, 42, 46, 47]. We recently observed that the frequency of cytosine methylation changes in wheat genome exerted by somatic hybridization was 23.6 % through the methylation-sensitive amplification polymorphism (MSAP) analysis [31], and was more intense than the frequency (13 %) exerted by allopolyploidization in a newly synthesized allohexaploid wheat [12]. This mechanism may therefore explain why C/T SNPs were more common in the SR3 *vs* JN177 comparison (2.07 per 1000 nt) than in the JN177 *vs* wheat database ESTs comparison (Table 2). Cytosine methylation polymorphisms are known to account for at least some of the differential gene transcription shown by SR3 and JN177 [48]; a further contributor may well be chromatin remodeling. The frequency of SNPs (18.17 and 19.21 per 1000 nt) and indels (0.95 and 0.92 per 1000 nt) induced SR3 involved genes participating in chromatin assembly/disassembly and nucleosome assembly (Table 6), consistent with the induction of widescale epigenetic changes resulting from somatic hybridization.

The evolutionary path followed by most polyploids is to reduce their genome size by deletion [9, 12, 42, 49–53]. After many generations of such activity, many of the sequences which have become duplicated as a result of the allopolyploidization event revert to single copy and the genome becomes increasingly diploid-like [9]. A good example of such a cryptic polyploid is maize [54, 55]. Some of the sequences lost in SR3 (compared to JN177) were of substantial length (Fig. 1b), indicating that the response to somatic hybridization also includes sequence deletion. On the other hand, the preference to large insertion was alleviated in SR3-JN177 alignment, and the preference to either insertion or deletion was not correlative to indel size (Fig. 1b). Consistently, AFLP and southern blotting outcomes found that the extent of sequence loss over generations from the products of somatic hybridization was relatively modest [31]. These point to a potential difference in the way that genomes respond to polyploidization as opposed to somatic hybridization. A major difference is that a *de novo* sexual hybrid harbors the full genomes of the donor and recipient, while in the asymmetric somatic hybrid, only fragments of the donor are retained from the outset, and many are presumably eliminated prior to the regeneration from tissue culture. Based on this, the suggestion is that the potential disruptive effect of donor DNA is possibly much less.

Asymmetric somatic hybridization was performed between UV-irradiated tall wheat grass protoplast and unirradiated wheat protoplast, both of which were produced from calli. During this process follows mutagenic factors including protoplast formation, UV irradiation, callus induction, and plant regeneration [3]. We found that these mutagenic factors had substantially slight effect on the genetic variation of SR3 and other other derivatives of wheat-based somatic hybrids by a set of molecular biomarker assays and comparing glutenin genomic sequences [31, 32]. Therefore, it could be concluded that the high frequency of genetic variation majorly comes from the genomic shock during asymmetric somatic hybridization.

### Putative mechanisms underlying indel formation induced by somatic hybridization

Numerous indels differentiate the genomes of SR3 and JN177 (Table 5). The GC content of the indels identified in the SR3 *vs* JN177 comparison was comparable to that in the indels differentiating the JN177 unigenes from those present in other wheat. As a result, it is not tenable to suggest that indels induced by the somatic hybridization process reflect a particular level of GC content. The proposition that indel formation, along with the other genomic changes induced by allopolyploidization, is non-random [56] does however require that some feature of the local sequence acts as a recognition feature. The GC content of the nucleotide 5′ adjacent only to the indels differed from that of other nucleotides in flanking sequence (Fig. 2), which does suggest that GC content could be a candidate signal for indel formation. Note that the GC content of the sequences adjacent to indels differed between the SR3 *vs* JN177 and the JN177 *vs* database wheat ESTs comparisons (Fig. 2), which implies that the mechanism underlying indel formation during somatic hybridization may well be distinct from that controlling the process in a sexual hybrid. Repetitive sequences are particularly susceptible to expansion and contraction, and we found 76 indels in the SR3 *vs* JN177 comparison harbored repetitive sequences in their flanking sequence (Fig. 3b, e and Additional file 5: Figure S2C, D). Repetitive sequences flanking indels may serve as recombination sites to promote indel formation via homologous recombination, because double strand breaks (DSBs) appear to be common in regions enriched for repetitive sequences, and these “fragile sites” represent hotspots for recombination [57].

### The possible association between sequence variation and phenotype in SR3

The genetic variation was found to affect gene expression or the activity of gene production [9–20], therefore lead to phenotypic alteration. Numerous transcriptomic and proteomic differences have been established between SR3 and JN177 [28, 29, 58]. Here, it was revealed that a substantial number of unigenes (involved in a range of plant processes) harbored SNPs and/or indels between SR3 and JN177 (Table 6, Additional file 3 and 4: Tables S2 and S3). Especially, the high salt tolerance of SR3 is closely associated with its superior ability of antioxidation and redox homeostasis maintenance [28, 30], and here the unigenes participating in cell redox homeostasis and response to stress and oxidative stress also has higher SNP and indel frequencies in SR3 (Additional file 4: Table S3). Moreover, EST sequences that absolutely matched to the probes of cDNA array with differential expression profiles [58] possessed some SNPs and indels, and some of these sequence variants could well contribute to the phenotypic difference between SR3 and JN177 (Additional file 7: Table S4). One established example is the gene *TaCHP*, which is more strongly transcribed in SR3 than in JN177 and is associated with SR3’s salinity tolerance [33]. The SR3 *TaCHP* allele differs from that present in JN177 by a deletion in its promoter sequence. A second example concerns *TaSRO1*, also associated with the salinity tolerance of SR3; the SR3 and JN177 alleles differ by three SNPs, one of which is responsible for the higher activity of the SR3 allele [30]. The sequences of the SR3 and JN177 alleles of *TaFLS1* (likewise associated with salinity tolerance) have been shown to be differentially methylated, and this polymorphism can account for their differential transcript abundance [48]. The headline conclusion to be drawn from this analysis is that asymmetric somatic hybridization can induce a high frequency of both genetic and epigenetic variation, some of which is likely responsible for alterations in the phenotype.

During standard somatic hybridization, the genomes of biparents emerge together, which induces drastic genomic shock and therefore leads to large scale of genome rearrangement, sequence elimination and genetic variation [3]. As a result, the hybrids often exhibit development dysfunction and sterility [3]. For asymmetric somatic hybridization, the protoplasts of donor (tall wheatgrass) are irradiated and its genome is fragmented, so in the fused cells of donor and recipient (wheat), most of chromatin fragments of donor are eliminated and a very few fragments are introgressed into the genome of recipient, while the genome of recipient has almost no structural change [3]. This event induces moderate genomic shock and results in bearable genetic variation. Therefore, the hybrids are fertile and their wheat derivatives often exhibit diverse phenotypes such as high salt and drought tolerance, superior stripe rust resistance, as well as high quality and yield traits (low stem, big ear, big grain, and high yield, etc.) [3, 5, 25, 26], which can be selected to breed cultivar with improved agricultural traits.

## Methods

### Stress treatment and cDNA library construction

Given that SR3 has demonstrated improved levels of tolerance to both salinity and drought, we proposed to compare the sequence variation of ESTs that are produced under the control and after exposure to saline and osmotic stresses to gain the association between genetic variation and abiotic stress tolerance. Three-leaf-stage seedlings of both cv. SR3 and cv. JN177 were exposed to half strength Hoagland’s liquid medium containing either 200 mM NaCl for 0, 0.5, 1, 12, 24, 48 or 72 h, or to 18 % PEG for 0, 0.5, 1, 12, 24 or 48 h. RNA was extracted from both roots and leaves using the Trizol reagent (Invitrogen, USA), and purified using an OligotexTM-dT_30_ mRNA Purification Kit (TaKaRa, Japan). Equimolar amounts of the various RNA samples were mixed to obtain both an SR3 and a JN177 mRNA pool, which formed the template for the construction of two cDNA libraries using a CloneMiner™ cDNA Library Construction Kit (Invitrogen, USA), according to the manufacturer’s protocol. Given that the genetic variation induced by asymmetric somatic hybridization was remarkably higher than that induced by other mutagenic factors protoplast formation, UV radiation, callus induction and plant regeneration [31, 32], other cDNA libraries reflecting the genetic variation of these mutagenic factors were not constructed.

### cDNA sequencing and sequence assembly

The two cDNA libraries were cultured on LB agar plates containing 50 mg/mL kanamycin overnight at 37 °C. The monoclones were selected randomly, and the inserts present sequenced based on the M13F primer by the Sanger sequencing method using ABI 3700 sequencer under the control of Sequencing Analysis 5.2.0 with the default parameters. The cDNAs were directionally inserted in the plastid, and the inserted sequences were sequenced from 5′-terminal using the M13F primer, which can increase the proportion of full-length cDNA to be sequenced. To ensure the quality of sequences, only 800 nt fragments from the reading start were selected for following analysis. Plastid and adapter sequences were removed to assume the sequencing quality of Q20 using the Seqman programme [59]. Low quality and short (<100 nt) sequences were removed with EGassemble (http://egassembler.hgc.jp). The quality of the cleaned sequences was further checked via randomly selecting more than 2000 sequences to analyze sequencing peak graph. The edited sequences were assembled using CAP3 software to produce a set of contigs and singletons (overlap 50 nt, identity 95 %) [60].

### Local BLAST and annotation

The resulting contig and singleton sequences were subjected to a BLASTn search, applying an E-value cut-off of 1E-10 and an HSP length cut-off adjusted to 33, to the EST sequences of bread wheat, *T. monococcum* (modern cultivated diploid A genome carrier), *Ae. tauschii* (diploid D genome progenitor species), *T. turgidum* (BA tetraploid progenitor species) and *Ae. speltoides* (diploid related to the B genome ancestor) mounted in GenBank (http://www.ncbi.nlm.nih.gov/genbank/dbest) (download date: Dec 12, 2014). A BLASTx search of the non-redundant protein database (ftp://ftp.ncbi.nlm.nih.gov/blast/db/) was used predict the full length cDNA sequences, where the matched EST sequence possessing encoding sequence of N-terminal amino acid (containing start codon ATG) was defined as full length cDNA. The SR3 and JN177 sequences were also subjected to a BLASTx search of the Arabidopsis cDNA database (ftp://ftp.arabidopsis.org/home/tair/Sequences/ATH_cDNA_EST_sequences_FASTA/ATH_cDNA_sequences_20101108.fas), applying an E-value cut-off of 1E-10 and an HSP length cut-off adjusted to 33. Matching sequences were classified by gene ontology using the TAIR GO annotation tool (http://www.arabidopsis.org/tools/bulk/go/index.jsp) and processed for GO classification and pathway enrichment using Blast2GO [61] using the entire GO-annotated collection of SR3 and JN177 ESTs as the reference.

### Polymorphism calculation

The SNP pattern was defined as the nucleotide of query sequence (e.g., A) with the nucleotide of subject sequence (e.g., G) as the reference (SNP was G → A). The insertion and deletion were also defined with the subject sequence as the reference. The sequences with BLASTn identity > 96 % were selected for calculation SNP and indel frequencies to exclude the interference of paralogous genes as possible [62], which were calculated as the ratio of total SNP and indel amounts to the total length of matched regions of all ESTs with identity > 96 %, respectively. The matched coverage (>96 % identity coverage) was defined as the ratio of the total length of all matched regions of ESTs with identity >96 % to the total length of these ESTs. To analyze the possible association between genetic variation and transcriptional pattern, the sequences were subject to BLASTn against the sequences (60 nt) of differentially expressed probes of wheat cDNA array in our previous study exploring the transcriptomic difference between SR3 and JN177 seedlings under the control and after exposure to NaCl and PEG treatments [58]. The unigenes that absolutely matched probe sequences were selected for calculating SNPs and indels.

## Conclusions

Asymmetric somatic hybridization induces high frequency of point mutations and indels, with a stronger intense than the genetic variation during allopolyplodization, which is the molecular basis of the phenotypic alteration of somatic hybrids. Moreover, the genetic variation induced by asymmetric somatic hybridization has different characteristics from those induced by allopolyplodization, demonstrating the specific genomic evolution in somatic hybrids. Therefore, asymmetric somatic hybridization provides appropriate material to comprehensively explore the nature of the genetic variation induced by genomic shock.

Sequences used for analysis in this work were submitted to Genbank (Accession number: JZ881292 - JZ892704).

## Additional files

Additional file 1: Table S1. Details of the cDNA libraries and the contig assembly. (DOCX 11 kb) Additional file 2: Figure S1. The distribution of unigene sequences. (TIFF 943 kb) Additional file 3: Table S2. SNP and indel frequencies in unigenes participating in metabolic processes. (DOCX 14 kb) Additional file 4: Table S3. SNP and indel frequencies in unigenes participating in other processes. (DOCX 14 kb) Additional file 5: Figure S2. Characterization of indels. (A) An insertion (291–764) present in an SR3 unigene has flanking sequence which matches that in its JN177 homolog. (B) A deletion (490–523) present in an SR3 unigene has flanking sequence which matches that in its JN177 homolog. (C) An insertion (143–320) present in an SR3 unigene, which harbors a run of G’s (shown in green) in both flanking sequences. (D) A deletion (664–747) present in an SR3 unigene which harbors a CATCCC repeat (shown in green) in both flanking sequences. (E) A deletion (729–868) present in an SR3 unigene, in which the identity of three nucleotides (676–678) differ between the SR3 and JN177 homologs. (TIFF 2567 kb) Additional file 6: Figure S3. Translocations and sequence chimeras induced by somatic hybridization. (A) In the homologs SR3_2LCP192_G10 and JN177_firstas231, the identical sequence is found in positions 1187–1367 in the former allele, but at 1–181 in the other. SR3_2LCP192_G10 also harbors a large deletion with a repeat sequence CAAGAAGGA. (B) In SR3_firstas716, nucleotides 88–196 do not align with JN177_LCP139_D11 nucleotides 157–278, but their terminal sequences are identical. (TIFF 1660 kb) Additional file 7: Table S4. Sequence variation for cDNAs which have been shown to be differentially transcribed in SR3 and JN177. ^a^: The treatments were conducted in our previous transcriptomic analysis (Liu et al., Plant Mol Biol, 2012;78:159–69). ^b^: The patterns of differential expressed probes in cDNA microarray (Liu et al., Plant Mol Biol, 2012;78:159–69). “up-regulated”: unigenes which were up-regulated in SR3, “down-regulated”:unigenes which were up-regulated in SR3. Control: half strength Hoagland’s liquid medium, PEG 0.5 h: seedlings exposed to 30 % PEG6000 for 0.5 h, NaCl 0.5 h and 24 h:seedlings exposed to 345 mM NaCl for 0.5 h and 24 h, respectively. ^c^: The number of unigenes that absolutely matched the sequences of probes. (DOCX 14 kb)
