# Supplementary material for: Asymmetric somatic hybridization induces point mutations and indels in wheat
Source: BMC Genomics. 2015 Oct 17;16:807. doi: 10.1186/s12864-015-1974-6 (PMC4609470; doi:10.1186/s12864-015-1974-6)
Supplement: Additional file 1: Table S1. — Details of the cDNA libraries and the contig assembly. (DOCX 11 kb) [file 12864_2015_1974_MOESM1_ESM.docx]

Supplementary table S1. Details of the cDNA libraries and the contig assembly

| cDNA library | Total EST sequences | Contigs | Singletons | Unigenes | Full-length | GC content  (%) |
| --- | --- | --- | --- | --- | --- | --- |
| SR3 | 18192 | 2097 | 7537 | 9634 | 4825 | 53.85 |
| JN177 | 9770 | 1207 | 5900 | 7107 | 2975 | 55.46 |
