# Supplementary figures and images for: Asymmetric somatic hybridization induces point mutations and indels in wheat
Source: BMC Genomics. 2015 Oct 17;16:807. doi: 10.1186/s12864-015-1974-6 (PMC4609470; doi:10.1186/s12864-015-1974-6)

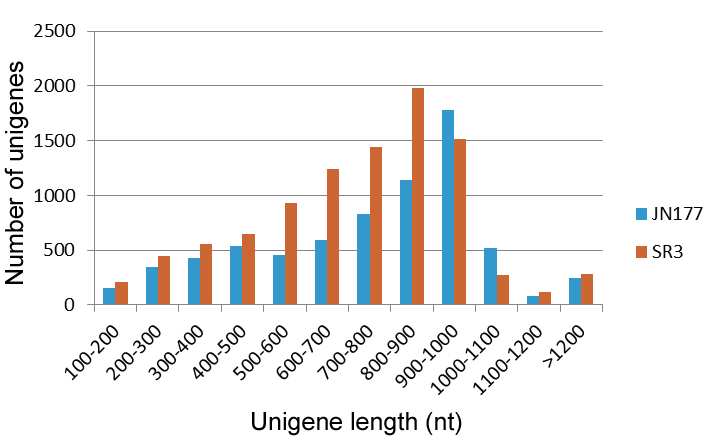

Supplement: Additional file 2: Figure S1. — The distribution of unigene sequences. (TIFF 943 kb) [file 12864_2015_1974_MOESM2_ESM.tif]

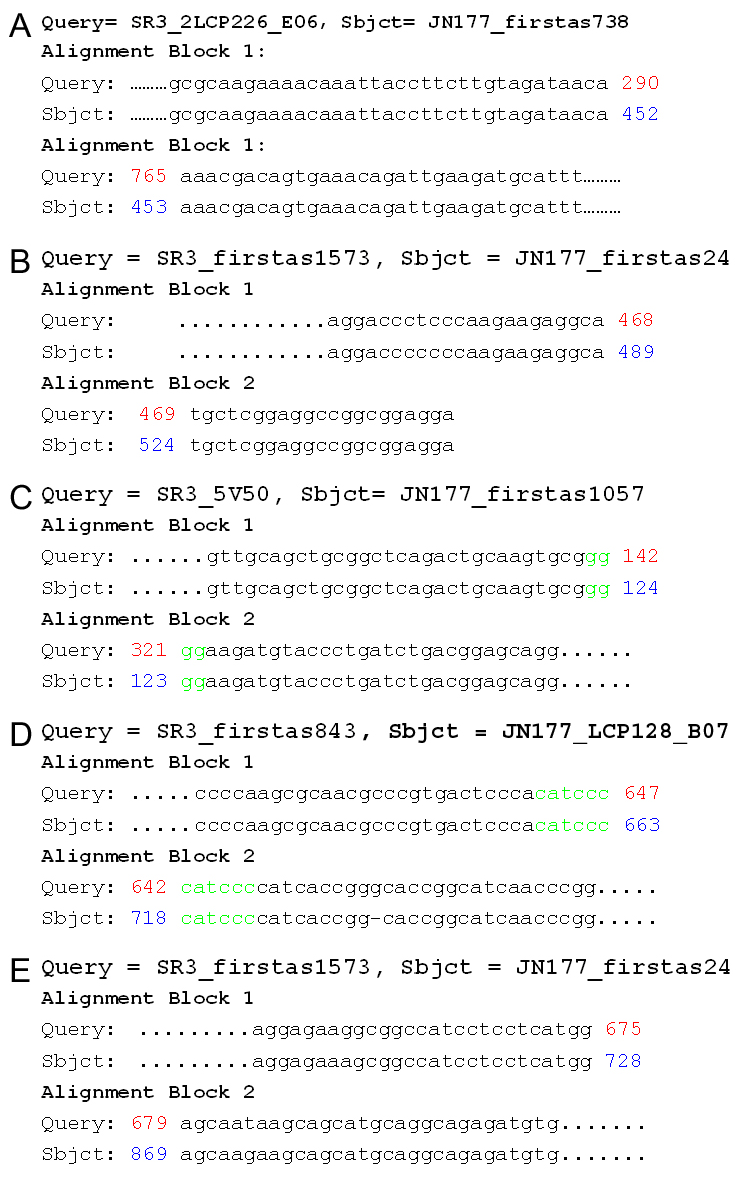

Supplement: Additional file 5: Figure S2. — Characterization of indels. (A) An insertion (291–764) present in an SR3 unigene has flanking sequence which matches that in its JN177 homolog. (B) A deletion (490–523) present in an SR3 unigene has flanking sequence which matches that in its JN177 homolog. (C) An insertion (143–320) present in an SR3 unigene, which harbors a run of G’s (shown in green) in both flanking sequences. (D) A deletion (664–747) present in an SR3 unigene which harbors a CATCCC repeat (shown in green) in both flanking sequences. (E) A deletion (729–868) present in an SR3 unigene, in which the identity of three nucleotides (676–678) differ between the SR3 and JN177 homologs. (TIFF 2567 kb) [file 12864_2015_1974_MOESM5_ESM.tif]

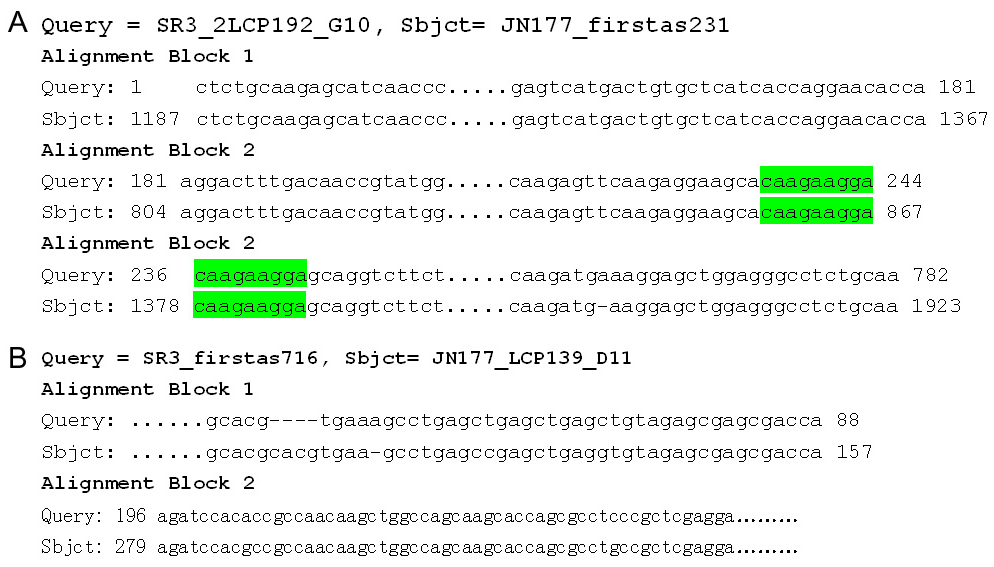

Supplement: Additional file 6: Figure S3. — Translocations and sequence chimeras induced by somatic hybridization. (A) In the homologs SR3_2LCP192_G10 and JN177_firstas231, the identical sequence is found in positions 1187–1367 in the former allele, but at 1–181 in the other. SR3_2LCP192_G10 also harbors a large deletion with a repeat sequence CAAGAAGGA. (B) In SR3_firstas716, nucleotides 88–196 do not align with JN177_LCP139_D11 nucleotides 157–278, but their terminal sequences are identical. (TIFF 1660 kb) [file 12864_2015_1974_MOESM6_ESM.tif]
