# Supplementary material for: Asymmetric somatic hybridization induces point mutations and indels in wheat
Source: BMC Genomics. 2015 Oct 17;16:807. doi: 10.1186/s12864-015-1974-6 (PMC4609470; doi:10.1186/s12864-015-1974-6)
Supplement: Additional file 3: Table S2. — SNP and indel frequencies in unigenes participating in metabolic processes. (DOCX 14 kb) [file 12864_2015_1974_MOESM3_ESM.docx]

Supplementary table S2. SNP and indel frequencies in unigenes participating in metabolic processes

| Process | Unigene number | |  | SNP (per 1,000 nt) | | |  | InDel (per 1,000 nt) | | |
| --- | --- | --- | --- | --- | --- | --- | --- | --- | --- | --- |
|  | JN1a77 | SR3 |  | SR3-JN177 | SR3-Ta | JN177-Ta |  | SR3-JN177 | SR3-Ta | JN177-Ta |
| carbohydrate metabolic process | 304 | 459 |  | 10.45 | 6.22 | 5.97 |  | 1.81 | 1.41 | 1.00 |
| glycolysis | 58 | 110 |  | 11.23 | 5.63 | 6.11 |  | 1.95 | 1.70 | 1.10 |
| tricarboxylic acid cycle | 21 | 35 |  | 9.16 | 5.28 | 4.68 |  | 1.65 | 1.33 | 0.85 |
| nucleobase, nucleoside, nucleotide and nucleic acid metabolic process | 285 | 622 |  | 11.14 | 6.56 | 6.11 |  | 1.49 | 1.29 | 0.90 |
| ubiquitin-dependent protein catabolic process | 30 | 101 |  | 13.81 | 6.23 | 5.69 |  | 1.76 | 1.44 | 0.83 |
| lipid metabolic process | 110 | 240 |  | 14.25 | 6.46 | 5.67 |  | 1.41 | 1.33 | 0.92 |
| phosphate metabolic process | 219 | 301 |  | 11.39 | 7.21 | 6.29 |  | 1.61 | 1.28 | 0.89 |
| metabolic process | 2229 | 3531 |  | 12.38 | 6.35 | 5.91 |  | 1.54 | 1.29 | 0.89 |
| biosynthetic process | 942 | 1603 |  | 12.73 | 6.30 | 5.70 |  | 1.49 | 1.27 | 0.87 |
| phenylpropanoid metabolic process | 23 | 52 |  | 10.19 | 7.48 | 6.03 |  | 1.26 | 1.42 | 0.81 |
| protein metabolic process | 742 | 1158 |  | 12.50 | 6.12 | 5.45 |  | 1.53 | 1.24 | 0.83 |
| macromolecule metabolic process | 963 | 1635 |  | 12.09 | 6.15 | 5.59 |  | 1.52 | 1.24 | 0.83 |
| primary metabolic process | 1481 | 2461 |  | 12.12 | 6.26 | 5.78 |  | 1.57 | 1.29 | 0.90 |
