# Supplementary material for: Asymmetric somatic hybridization induces point mutations and indels in wheat
Source: BMC Genomics. 2015 Oct 17;16:807. doi: 10.1186/s12864-015-1974-6 (PMC4609470; doi:10.1186/s12864-015-1974-6)
Supplement: Additional file 4: Table S3. — SNP and indel frequencies in unigenes participating in other processes. (DOCX 14 kb) [file 12864_2015_1974_MOESM4_ESM.docx]

Supplementary table S3. SNP and indel frequencies in unigenes participating in other processes

| Process | Unigene number | |  | SNP (per 1,000 nt) | | |  | InDel (per 1,000 nt) | | |
| --- | --- | --- | --- | --- | --- | --- | --- | --- | --- | --- |
|  | JN177 | SR3 |  | SR3-JN177 | SR3-Ta | JN177-Ta |  | SR3-JN177 | SR3-Ta | JN177-Ta |
| protein folding | 76 | 128 |  | 12.17 | 6.27 | 5.56 |  | 1.84 | 1.43 | 1.02 |
| proteolysis | 143 | 244 |  | 12.15 | 6.21 | 6.82 |  | 1.83 | 1.46 | 1.04 |
| transport biological process | 398 | 810 |  | 11.59 | 5.93 | 5.08 |  | 1.34 | 1.25 | 1.09 |
| signal transduction | 81 | 245 |  | 11.25 | 6.06 | 5.09 |  | 1.33 | 1.38 | 0.87 |
| protein transport | 89 | 203 |  | 9.42 | 6.04 | 4.49 |  | 1.30 | 1.35 | 0.98 |
| phosphorylation | 192 | 253 |  | 10.89 | 7.34 | 6.37 |  | 1.79 | 1.45 | 1.16 |
| cell redox homeostasis | 26 | 51 |  | 10.85 | 5.71 | 4.79 |  | 1.84 | 1.41 | 1.00 |
| regulation of cellular process | 224 | 580 |  | 10.81 | 5.99 | 5.57 |  | 1.50 | 1.37 | 0.92 |
| transmembrane transport | 197 | 272 |  | 11.84 | 6.33 | 4.97 |  | 1.35 | 1.28 | 1.23 |
| oxidation-reduction process | 503 | 807 |  | 12.96 | 6.23 | 6.19 |  | 1.61 | 1.41 | 1.07 |
| response to stress | 246 | 820 |  | 11.54 | 6.91 | 5.76 |  | 1.66 | 1.28 | 1.08 |
| response to oxidative stress | 101 | 173 |  | 12.07 | 7.19 | 6.69 |  | 1.66 | 1.38 | 1.03 |
