# Supplementary material for: Asymmetric somatic hybridization induces point mutations and indels in wheat
Source: BMC Genomics. 2015 Oct 17;16:807. doi: 10.1186/s12864-015-1974-6 (PMC4609470; doi:10.1186/s12864-015-1974-6)
Supplement: Additional file 7: Table S4. — Sequence variation for cDNAs which have been shown to be differentially transcribed in SR3 and JN177. a: The treatments were conducted in our previous transcriptomic analysis (Liu et al., Plant Mol Biol, 2012;78:159–69). b: The patterns of differential expressed probes in cDNA microarray (Liu et al., Plant Mol Biol, 2012;78:159–69). “up-regulated”: unigenes which were up-regulated in SR3, “down-regulated”:unigenes which were up-regulated in SR3. Control: half strength Hoagland’s liquid medium, PEG 0.5 h: seedlings exposed to 30 % PEG6000 for 0.5 h, NaCl 0.5 h and 24 h:seedlings exposed to 345 mM NaCl for 0.5 h and 24 h, respectively. c: The number of unigenes that absolutely matched the sequences of probes. (DOCX 14 kb) [file 12864_2015_1974_MOESM7_ESM.docx]

Supplementary table S4. Sequence variation for cDNAs which have been shown to be differentially transcribed in SR3 and JN177

| Treatment ^a^ | Expression ^b^ | Sequences ^c^ | SNPs |  |  | Small indels (1-10 nt) | |  | Large indels (>21 nt) | |  |
| --- | --- | --- | --- | --- | --- | --- | --- | --- | --- | --- | --- |
|  |  |  | Transition | Transversion | Total | Insertion | Deletion | Indel | Insertion | Deletion | Indel |
| Control | Up-regulated | 17 | 16 | 14 | 17 | 11 | 11 | 13 | 1(24) | 1(414) | 2 |
|  | Down-regulated | 42 | 37 | 37 | 39 | 23 | 13 | 27 | 0 | 0 | 0 |
| PEG0.5h | Up-regulated | 10 | 10 | 10 | 10 | 5 | 6 | 8 | 0 | 0 | 0 |
|  | Down-regulated | 21 | 19 | 20 | 21 | 13 | 10 | 15 | 0 | 0 | 0 |
| NaCl0.5h | Up-regulated | 31 | 27 | 28 | 30 | 16 | 18 | 23 | 1(24) | 1(414) | 2 |
|  | Down-regulated | 4 | 1 | 3 | 3 | 0 | 0 | 0 | 0 | 0 | 0 |
| NaCl24h | Up-regulated | 35 | 29 | 27 | 31 | 20 | 16 | 21 | 1(24) | 0 | 1 |
|  | Down-regulated | 24 | 21 | 21 | 22 | 14 | 8 | 17 | 0 | 0 | 0 |
| Total | Up-regulated | 2 | 2 | 2 | 2 | 1 | 1 | 1 | 0 | 0 | 0 |
|  | Down-regulated | 2 | 1 | 2 | 2 | 0 | 0 | 0 | 0 | 0 | 0 |

^a^: The treatments were conducted in our previous transcriptomic analysis (Liu et al, Plant Mol Biol, 2012, 78: 159-69).

^b^: The patterns of differential expressed probes in cDNA microarray (Liu et al, Plant Mol Biol, 2012, 78: 159-69). “up-regulated”: unigenes which were up-regulated in SR3, ”down-regulated”:unigenes which were up-regulated in SR3. Control: half strength Hoagland's liquid medium, PEG 0.5h: seedlings exposed to 30% PEG6000 for 0.5h, NaCl 0.5h and 24h:seedlings exposed to 345mM NaCl for 0.5 h and 24 h, respectively.

^c^: The number of unigenes that absolutely matched the sequences of probes.
